# Time-Dependent Changes in Malondialdehyde and Free-Hemoglobin in Leukoreduced and Non-Leukoreduced Canine Packed Red Blood Cells Units During Storage

**DOI:** 10.3390/vetsci12090838

**Published:** 2025-08-30

**Authors:** Arianna Miglio, Aurora Barbetta, Valentina Cremonini, Olimpia Barbato, Giovanni Ricci, Valeria Toppi, Luca Avellini, Valentina Cavani, Maria Teresa Antognoni

**Affiliations:** Department of Veterinary Medicine, University of Perugia, 06123 Perugia, Italyolimpia.barbato@unipg.it (O.B.); luca.avellini@unipg.it (L.A.);

**Keywords:** malondialdehyde, free hemoglobin, leukoreduction, CPD-SAGM, oxidative stress, blood storage, storage lesions, transfusion medicine

## Abstract

Storage of Blood units determines the accumulation of harmful substances, such as malondialdehyde (MDA) and free hemoglobin (fHb). These may lead to several complications, including cardiovascular, neurodegenerative, and metabolic disorders in recipients. The objective of this study was to evaluate the concentrations of MDA and fHb in canine leukoreduced (LR) and non-leukoreduced (NLR) packed red blood cells (pRBC) during the storage period of six weeks. Blood samples were collected from six healthy adult Weimaraner dogs (three females and three males). Whole blood was stored in citrate-phosphate-dextrose saline-adenine-glucose-mannitol additive solution (CPD-SAGM) bags and, for each donor, two pRBC units (one NLR and one LR) were produced and stored at 4 °C for 42 days. Samples were collected on days 0, 7, 14, 21, 28, 35, and 42, and analyzed for malondialdehyde (MDA) using a canine-specific ELISA method, and for free hemoglobin (fHb) using the Harboe direct spectrophotometric method. The results demonstrated a statistically significant reduction in MDA accumulation in LR-pRBC compared to NLR-pRBC blood units and lower values of fHb in LR at T6. However, no significant difference in fHb levels were demonstrated. These findings suggest that leukoreduction may limit oxidative stress during blood storage, reducing the potential adverse effects of transfusions related to oxidative damage.

## Simple Summary

During the storage of blood units, red blood cells are exposed to processes such as oxidative stress and hemolysis. The present study investigates the biochemical differences between leukoreduced and non-leukoreduced blood units, with a particular emphasis on the levels of malondialdehyde, a lipid peroxidation product, and free hemoglobin, an indicator of hemolysis in blood units collected from six healthy adult dogs over six weeks. In the fifth and sixth weeks, a statistically significant decrease in malondialdehyde levels was observed in the leukoreduced units compared to the non-leukoreduced units. However, no statistically significant difference was detected in free hemoglobin levels. This study demonstrated that leukoreduction should be applied also in veterinary medicine.

## 1. Introduction

Blood transfusion is a medical intervention often performed in emergency situations such as severe trauma, hemolytic anemia or acute bleeding, in both human and veterinary medicine [1,2,3]. Although the transfusion procedure is undoubtedly life-saving, it is important to note that adverse effects may occur, especially in high-risk patients [4,5,6,7]. In 2021, the Association of Veterinary Hematology and Transfusion Medicine (AVHTM) published the Transfusion Reaction Small Animal Consensus Statement (TRACS) which aimed to define, diagnose, monitor and prevent the main transfusion-related adverse effects [8,9]. In particular, the most important adverse effects are due to storage lesions, which derive to the accumulation of toxic substances and pro-oxidant molecules. These can both reduce osmotic fragility and decrease deformability of red blood cellular membranes inducing hemolysis in transfused blood and adverse reactions in the host [10,11,12,13]. It has been recently postulated that the development of storage lesions and the adverse effects of transfusion are caused mainly by leukocyte metabolites and reactive oxygen species (ROS), which can induce proinflammatory, prothrombotic and cytotoxic stimuli, as well as complement system activation [14,15,16,17].

With the aim of reduce storage lesions in stored erythrocytes and adverse reactions in the recipients, leukoreduction is widely used in human transfusion medicine [17,18]. In fact, this procedure allows to separates white blood cells and platelets from whole blood and packed red blood cell (pRBC) units by using a filter system. Unfortunately, this technique is not yet commonly applied in veterinary medicine [10,12,15,16,19].

Malondialdehyde (MDA), a by-product of lipid peroxidation derived from polyunsaturated fatty acids, has been observed to increase in cases of decreased antioxidant enzyme activity and increased ROS concentrations. In humans, high MDA levels have been documented in various chronic and acute disorders, including cardiovascular, neurodegenerative, metabolic, and infectious diseases [20,21,22,23]. As MDA is the major product of lipid peroxidation, it is frequently used as an oxidative stress marker in studies regarding transfusion of stored blood in humans [21,24,25]. In veterinary medicine, only a few studies have been conducted on this topic [13,26], evaluating MDA concentrations during the storage of not-processed whole blood units in dogs and donkeys, without investigating the possible influence of leukoreduction.

Since oxidative stress is one of the primary causes of hemolysis with hemoglobin release during the shelf-life of blood bags [27], the concentration of free hemoglobin (fHb) has been proposed as a useful marker of storage lesions in humans [11,24,28,29,30,31,32] and, only recently, in veterinary medicine [12,13,14,33,34]. The presence of fHb in stored blood is due to the rupture of erythrocytes, a phenomenon strongly influenced by the decrease in the 2,3-diphosphoglycerate (2,3-DPG) and ATP levels. These depletions decrease deformability and oxygen-carrying capacity of RBC leading to morphological and rheological changes [35,36,37]. Interestingly, elevated in vivo or ex vivo fHb concentrations in humans have been associated with an increased risk of sepsis [38], promoting growth of Streptococcus pneumoniae [39], nephrotoxicity and acute kidney injury [40], acute lung injury, multiple organ failure, and increased risk of death [36,41]. Moreover, fHb significantly contributes to the production of free radicals via autoxidation and Fenton reactions, induces inflammation, and impairs vascular function largely through nitric oxide depletion [42,43].

The benefits of leukoreduction have not been completely investigated in veterinary transfusion medicine, and no study has been conducted to date to assess its impact on the contemporary variation of the oxidative rate and hemolysis in canine paked Red Blood Cells units (pRBC). The aim of this study was to investigate the effect of leukoreduction on the time-dependent changes of MDA and fHb concentrations in canine pRBC units during storage, to improve knowledge on this topic.

## 2. Materials and Methods

### 2.1. Animal Selection

Six adult Weimaraner dogs, three females and three males, were enrolled in this study. All dogs were breads all from the same breeder and were subjected to the same stabling regimen. All dogs were also fed the same maintenance diet, consisting of commercial food with a medium fat content. Subjects were evaluated as clinically healthy based on physical examination and routine biochemical and hematological analysis (complete blood count, biochemical profile including liver, kidney, protein, and electrolyte profiles, and serological testing for *Leishmania infantum*, *Ehrlichia canis*, *Babesia* spp., *Rickettsia* spp., *Anaplasma phagocytophilum*, and *Dirofilaria immitis*) at the Veterinary Teaching Hospital of the University of Perugia (PG-VTH). All the subjects met the requirements set by the Italian Ministry of Health guidelines regarding transfusion medicine [44]. In particular, all donors aged between 2 and 8 years, weighed between 27 and 35 kg with a body condition score of 4–5/9, were free from any current or previous diseases and were regularly treated for endo- and ectoparasites. All dogs tested negative for all serological tests. Appropriate informed consent was obtained from the owners for the enrollment.

### 2.2. Blood Collection, Processing and Storage

Blood was collected from each donor into citrate-phosphate-dextrose saline-adenine-glucose-mannitol additive solution (CPD-SAGM) bags. For each dog, 400 mL of blood was aseptically collected from the jugular vein using a commercial closed collection system (IMUFLEX CRC Blood Bag System, TERUMO PENPOL Ltd., 695003 Kerala, India). This system comprises four bags connected in series, one containing the anticoagulant citrate-phosphate-dextrose (CPD) for whole blood collection, one containing the saline-adenine-glucose-mannitol (SAGM) additive solution, and two empty bags for plasma separation and leukoreduction. Each collected bag was centrifuged within an hour of collection at 3068 RCF (g) for 20 min at 4 °C (Rotixa 50 RS, Hettich Italia s.r.l., Milan, Italy). Plasma was then mechanically removed and transferred through the tubing to the attached transfer bag. To the pRBC thus obtained, the SAGM solution present in another satellite bag was added. At this point, half of the initial blood volume was subjected to leukoreduction by draining it through the leukoreduction filter integrated into the blood bag system, and then collected by gravity in a satellite bag; this procedure was completed for each bag within 2.5 h of collection. The remaining sample represented the non-leukoreduced one (NLR). Two pRBC units were therefore obtained for each dog: one leukoreduced (LR) and one NLR. All the blood units were finally stored in a blood bank refrigerator at 4 °C for six weeks (42 days).

### 2.3. Samples Collection

Two aliquots of 3 milliliters each were aseptically collected in anticoagulant-free tubes from either NLR and LR blood bags at day 0 (T0), day 7 (T1), day 14 (T2), day 21 (T3), day 28 (T4), day 35 (T5) and day 42 (T6). All samplings were carried out from blood bags after gently resuspending the corpuscular fraction and under a laminar flow hood to ensure sterile working conditions. At T0, a fresh whole blood sample was also collected directly from the donors immediately before donation to obtain plasma donor samples. All the aliquots were centrifuged at 1811 RCF (g) for 10 min at 4 °C and the supernatant was then processed for Harboe and malondialdehyde (MDA) tests.

### 2.4. Malondialdehyde (MDA) Method

MDA levels were assayed using specific canine ELISA development systems (AssayGenie, Germany; Catalog N°: CNEB0405), following the manufacturer’s instructions. All steps were performed in duplicate and at room temperature. The MDA assay sensitivity was <0.088 nmol/mL and the assay range were between 0.312 and 20 nmoL/mL. The spike average recovery was 93%. The intra- and inter-assay reproducibility were ≤5.4% and ≤7.8% respectively.

### 2.5. Harboe Direct Spectrophotometric Method

The Harboe method was used to measure oxyhemoglobin concentration by spectrophotometric detection at 415 nm, with background correction based on absorbance readings at 380 nm (nonspecific plasma interferents) and 450 nm (bilirubin/albumin complexes), as previously described [45,46,47]. All samples were diluted 1:2 in distilled water. Absorbance values were validated within the linear range of the spectrophotometer (0–2 absorbance units). Hemoglobin concentrations were calculated according to the equation provided by Cookson et al. (2004) [47], applying a calibration coefficient (k) of 1, as described by Harboe (1959) [46] and Malinauskas et al. (1997) [45].

### 2.6. Statistical Analysis

Data obtained are presented as mean ± SD. Statistical analysis was performed using two-way analysis of variance (ANOVA) with post-hoc Tukey’s HSD and Bonferroni tests to determine differences within and between groups. All analyses were conducted using GraphPad Prism The 8th version (GraphPad Software, San Diego, CA, USA). A significance level of *p* ≤ 0.05 was considered for all statistical tests.

## 3. Results

The results for MDA and fHb concentrations are summarized in Table 1.

**Figure 1:**
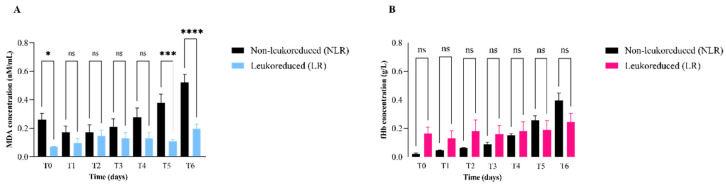
Time-course analysis of malondialdehyde (MDA; nM/mL) (**A**) and free hemoglobin (fHb; g/L) (**B**) concentrations in stored red blood cell units, comparing non-leukoreduced (NLR) and leukoreduced (LR) groups over a 42-day storage period. Samples were analysed at days 0 (T0), 7 (T1), 14 (T2), 21 (T3), 28 (T4), 35 (T5), and 42 (T6). Statistically significant differences between groups were determined by appropriate tests and are indicated by asterisks (* *p* < 0.05); “ns” indicates non-significant differences.

In particular, MDA levels were lower in the LR group compared to the NLR group throughout the storage time, with statistically significant higher values observed in NLR units on days 35 and 42 (*p* < 0.05) (Figure 1A). fHb concentrations remained stable in both groups throughout the storage time, with no statistically significant differences detected between LR and NLR units at any time point (Figure 1B).

**Figure 2:**
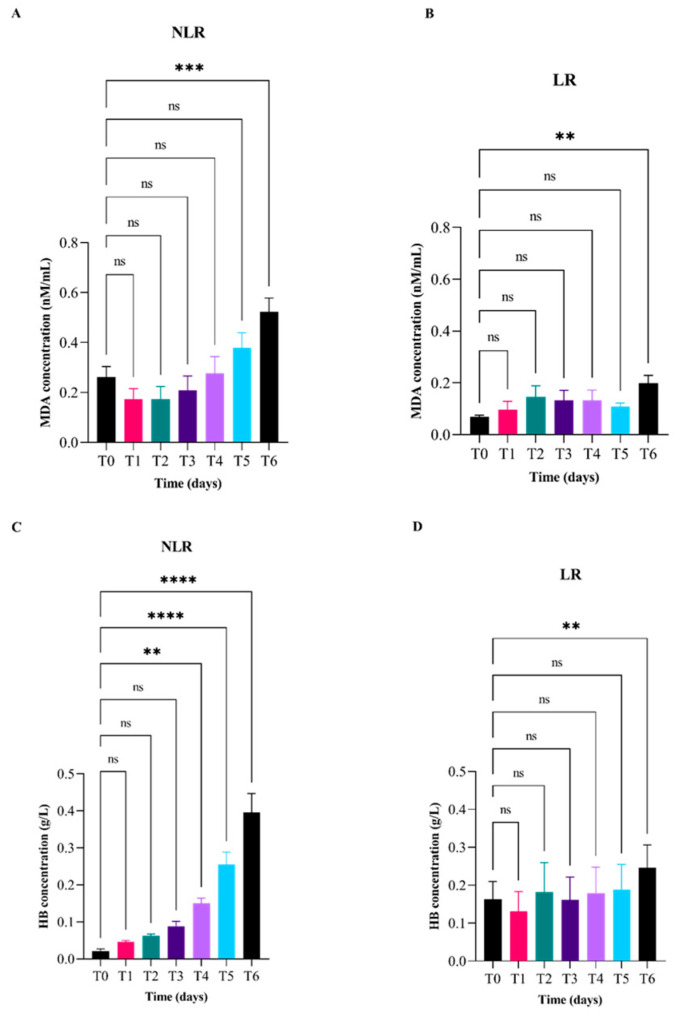
Temporal variation of malondialdehyde (MDA; top panel (**A**,**B**)) and free hemoglobin (fHb; bottom panel (**C**,**D**)) concentrations relative to baseline (day 0) in leukoreduced (LR) and non-leukoreduced (NLR) red blood cell units during 42 days of storage. Measurements were obtained at days 0 (T0), 7 (T1), 14 (T2), 21 (T3), 28 (T4), 35 (T5), and 42 (T6). Changes over time are expressed relative to day 0 values for each group. Statistically significant differences between groups were determined by appropriate tests and are indicated by asterisks (* *p* < 0.05); “ns” indicates non-significant differences.

The data depicted in Figure 2 represent the temporal changes in malondialdehyde (MDA; top panels A and B) and free hemoglobin (fHb; bottom panels C and D) concentrations, expressed relative to baseline levels (day 0), in LR and NLR red blood cell units over a 42-day storage period. A statistically significant increase in MDA concentration was observed in NLR units between day 0 and day 42 (Figure 2A). Specifically, MDA levels in LR units showed significant increase at day 14 and day 42 compared to baseline (Figure 2B). Regarding hemoglobin concentrations, NLR units exhibited statistically significant differences between day 0 and days 28, 35, and 42 showing a progressive increase. Interestingly, in LR units, a significant increase in hemoglobin concentration was only detected at day 42 compared to T0.

**Figure 3:**
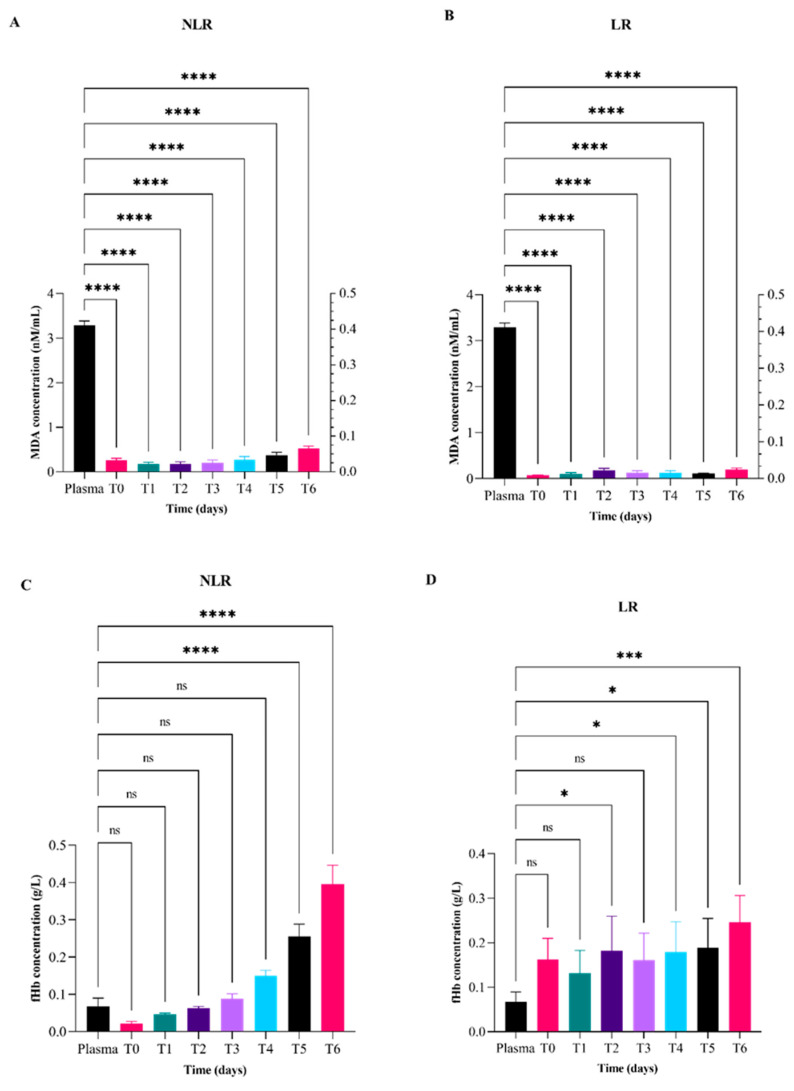
Temporal variation of malondialdehyde (MDA; top panel (**A**,**B**)) and hemoglobin (fHb; bottom panel (**C**,**D**)) mean values concentrations in leukoreduced (LR) and non-leukoreduced (NLR) pRBC units during 42 days of storage compared to mean values concentrations obtained from plasma donor samples. Measurements were obtained at days 0 (T0), 7 (T1), 14 (T2), 21 (T3), 28 (T4), 35 (T5), and 42 (T6). Changes over time are expressed relative to day 0 values for each group. Statistically significant differences were determined by appropriate tests and are indicated by asterisks (* *p* < 0.05); “ns” indicates non-significant differences.

The results reported in Figure 3 represent the comparison of malondialdehyde (MDA; top panels A and B) and free hemoglobin (fHb; bottom panels C and D) mean value concentrations in LR and NLR red blood cell units and plasma from the blood donor. Regarding MDA values, statistically significant differences were observed between plasma and all red blood cell units, both NLR and LR, at each time point (Figure 3A,B). Regarding free hemoglobin values, statistically significant differences compared to plasma were observed in NLR units after 35 and 42 days (Figure 3C). In the case of LR units, statistically significant differences with plasma were detected at days 14, 28, 35, and 42 (Figure 3D).

**Figure 4:**
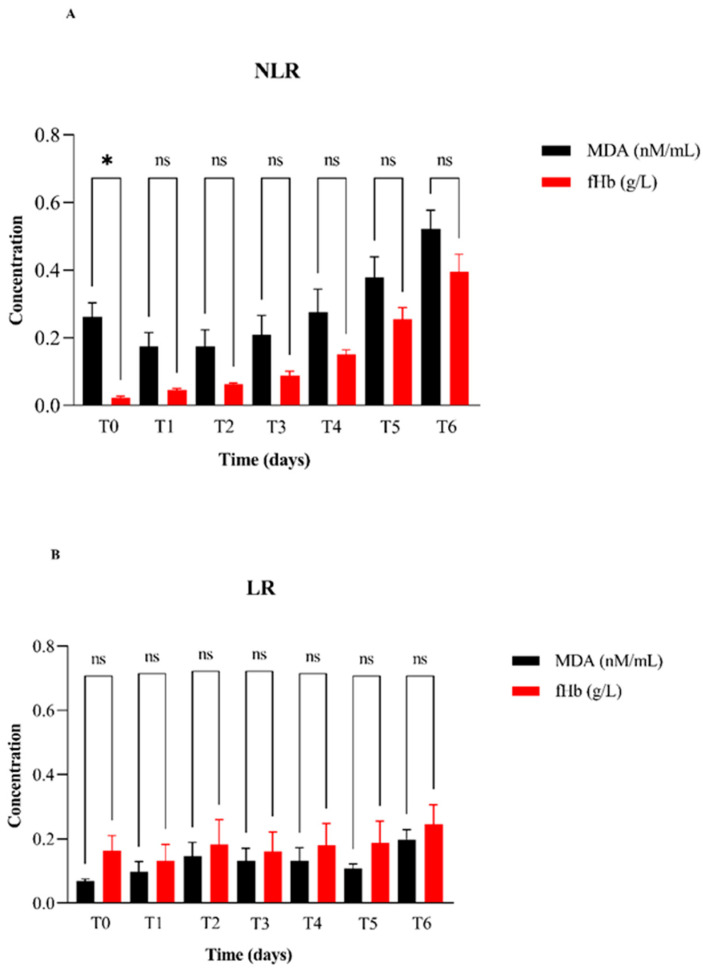
Comparison of relative concentrations of malondialdehyde (MDA) and free hemoglobin (fHb) in plasma between non-leukoreduced (NLR) (**A**) and leukoreduced (LR) (**B**) pRBC units over 42 days of storage. Measurements were taken on days 0 (T0), 7 (T1), 14 (T2), 21 (T3), 28 (T4), 35 (T5), and 42 (T6). Statistically significant differences between groups were determined by appropriate tests and are indicated by asterisks (* *p* < 0.05); “ns” indicates non-significant differences.

Finally, the results presented in Figure 4 provide a comparison of MDA and fHb concentration values between all NLR and LR pRBC units (Figure 4A,B). Specifically, Figure 4A shows a statistically significant difference between MDA and fHb concentrations only at T0 with MDA values markedly higher than fHb. Susprisingly, no statistically significant differences are observed at any time point in the leukoreduced units.

## 4. Discussion

This study represents the first attempt to compare free hemoglobin (fHb) and malondialdehyde (MDA) accumulation in leukoreduced (LR) and non-leukoreduced (NLR) blood units during storage in veterinary medicine, to improve the investigation regarding leukoreduction effect on stored blood units’ quality. Our results, showing a marked higher increase in MDA concentrations in NLR units compared to LR ones, clearly indicate that leukoreduction has a buffering effect on malondialdehyde production, probably modulating lipid peroxidation and oxidative stress. This result is congruent with data reported in humans, where different studies have documented that the presence of leukocytes in stored blood units is a crucial factor in triggering oxidative stress, since they release bioactive factors, such as enzymes, cytokines/chemokines and free radicals, causing damage to cell membranes during storage [17,48,49] and transfusion-related adverse effects [50,51]. Not least, in human medicine MDA has also a potential direct adverse effects, contributing to the development of cardiovascular disease and neurodegenerative and metabolic disorders [23].

The beneficial effect of pre-storage leukoreduction in canine blood units has already been discussed by some authors, emphasizing its role in reducing storage lesions and ameliorating blood units quality [10,52]. In a recent study Miglio et al. (2024) [10] used omics analysis to demonstrate the beneficial effect of leukoredution in canine pRBC showing more elevated levels of glycolytic metabolites, high energy phosphate compounds, and antioxidant metabolites, in LR compared to NLR blood units. These findings indirectly suggested a reduction in oxidative stress and in storage lesions development in canine LR pRBC.

The mean MDA plasma value obtained in our study is similar to those showed in healthy dogs in the limited literature available [53,54,55,56], even if a lot of these studies employed different, non-species-specific methodologies for the quantification of MDA, as the thiobarbituric acid reactive substances (TBARS assay). Notably, we used a specie-specific ELISA kit method with high sensitivity (reference values 0.312 to 20 nM/mL.) previously applied only by Nazzal et al. (2021) [56] who founded slightly lower values (0.9 ± 0.023 µmol/L) than ours.

In order to further investigate the starting condition of donors, in fact, we measured plasma MDA before donation and we then compared the results with those obtained in LR and NLR blood units during weekly samplings. Surprisingly, we found a mean plasma value (3.29 nM/mL, min 3.00 nM/mL max 3.61 nM/mL) considerably higher than those obtained at every time point in both LR and NLR groups, thus suggesting an immediate and long-lasting beneficial effect of additive solutions (citrate-phosphate-dextrose-adenine-1, i.e., CPDA-1, and SAGM) contained in pRBC blood bags as also discussed by other authors [57]. Particularly, SAGM is an adjuvant solution composed by sodium chloride, phosphate, adenine, guanosine, glucose and mannitol, with a pH of 5.7 only in pRBC units. In fact, this additive solution has the ability to support metabolic activity and osmoregulation, while protecting erythrocytes from oxidative damage [57]. Its presence and protective effect in pRBC blood units may have reduced MDA increase. For this purpose the importance of the use of SAGM as an additive solution is demonstrated by the only study who examined MDA changes at weekly intervals, for five weeks, in 10 canine whole blood bags using only CPDA-1 as anticoagulant additive solution [13], which found higher MDA values (from 10 to 20 μM/L) during all the storage period, compared with ours. In fact, in our study, MDA levels like these were never reached in both LR and NLR units. This finding, again, suggests other than a beneficial effect of the SAGM additive solution, a protective effect given by the removal of plasma, a source of inflammatory compounds such as cytokines, inflammatory proteins and reactive oxygen species (ROS) [49,58]. In veterinary medicine, only another study conducted on MDA concentrations during blood storage was performed in the donkey whole blood units stored in CPDA-1; consistent with our study, a gradual increase in this lipid peroxidation product was observed [26].

In our study, regarding fHb concentrations, the results obtained in NLR units follow a trend similar to what observed for MDA, revealing an increase in the hemolysis rate during storage. On the other side, fHb concentrations in LR were instead constant. However, no significant differences ware observed between these two groups. This result appears to be in line with those illustrated by other authors in human [28,29] and veterinary medicine [12,13,14,34].

When analyzing singularly the time-points, however, interesting data regarding fHb concentrations emerge in our study. Particularly, at T6 (42 days), a high level of fHb (approximately 0.4 g/L) was observed in NLR units, indicating a significant extent of hemolysis processes. On the other sides, in the case of LR units, no significant changes were observed during storage with only a slightly increase until T6 whose value obtained (0.24 g/dL) was however lower than that obtained in the same time in NLR units.

When comparing fHb levels obtained in donor plasma with those found in NLR samples, higher values were found in plasma until day 21 (T3), whereas in subsequent time points, fHb values increased gradually in NLR units, exceeding the basal levels of the donors, and showing significant difference at T5 (35 days) and T6 (42 days). Unfortunately, high concentrations of fHb in the donor samples can be caused by complicated blood sampling in the radial vein and/or by the sampling technique performed by using smaller caliber vessels and smaller gauge needles than that applied for blood donation. In contrast, in LR samples, fHb values were generally higher than in donor plasma in all time points, remaining steady during the storage at approximatively 0.20 g/dL. These high concentrations of fHb observed in LR units just at the beginning of their storage, could be explained with the mechanical damage resulting from the leukoreduction filter used [59] during pre-storage processing of blood units. Neverthless, the values showed in LR units during storage are reached in the NLR group only at T4 (28 days). From this time-point, the fHb values obtained in NLR units increased, reaching higher concentrations at the end of their shelf-life (T6, days 42), even if not significant. These data could suggest that leukoreduction probably could have mitigated hemolysis during the storage in LR units.

Our data reveal a trend of fHb concentration in NLR units similar to that showed in literature [12,13,14,52,60], but different as regards LR units [12,14,52,61]. In fact, although using different methods to detect and quantify hemolysis, all the previous studies that applied leukoreduction, showed a progressive increase of hemolysis, even if only Avenik et al. (2021) [52] identified a significant difference between LR and NLR units during storage. This discrepancies observed in LR units could be due to both the initial higher concentration of fHb identified, that could have hidden a slight progressive increase of hemolysis in our study, and the use of different additive solutions and filter system in blood bags as well as different techniques to measure hemolysis in samples.

The main limitation of this study was the small number of subject enrolled. However, we attempt to reduce this limit by enrolling dogs coming from the same breed, homogeneously divided by sex, and undergoing the same dietary regimen and breeding conditions. With this selection, we attempted to at least partially overcome the impact of possible donor-dependent variables. Another limitation of this study was the analysis of only two parameters during storage (fHb and MDA) which limits further consideration on oxidative stress damage in pRBC units stored for transfusion purposes. Other biomarkers indicative of oxidative stress during storage of blood units, such as reduced glutathione (GSH), cytokines and microparticles, even with the use of morphological and omics analysis of red blood cells, could be investigated in future studies to implement and consolidate the observed data.

Overall, the results of this study further support the hypothesis that leukoreduction has positive effects on the preservation of canine pRBC, showing new data regarding MDA as a parameter of oxidative stress damage in stored blood units. It could be proposed as a promising procedure also for veterinary transfusion medicine, with possibly clinical benefits for canine recipients.

## 5. Conclusions

This study corroborates the beneficial effects of leukoreduction and storage in additive solutions such as CPDA-1 and SAGM on the shelf life of canine erythrocytes, particularly effective in limiting lipid peroxidation phenomena. Indeed, both of these factors contribute to the control of oxidative damage, thus preserving blood units from excessive storage injury and ensuring the safety of pRBC transfusions. The increase in fHb observed in LR units at T0, probably appears to be caused by erythrocytes passage through leukoreduction filter used. Therefore, this system, imported from human transfusion medicine, may not be entirely appropriate for processing canine blood and specie specific filters should be manufactured. However, further studies are needed to validate the safety and effectiveness of leukoreduction in canine pRBC units, particularly with regard to the preventing effect on the development of storage lesions in blood units and the preventive effect on potential adverse effects in recipients.

## Figures and Tables

**Table 1:** Minimum, maximum and mean values of MDA and fHb concentrations for the six blood units analyzed, over time.

Measurand		Plasma	T0 ^2^		T1 ^3^		T2 ^4^		T3 ^5^		T4 ^6^		T5 ^7^		T6 ^8^	
**MDA ^1a^** **(nMN/mL)**			NLR ^9^	LR ^10^	NLR	LR	NLR	LR	NLR	LR	NLR	LR	NLR	LR	NLR	LR
	Media	3.29	0.26	0.10	0.20	0.10	0.17	0.17	0.21	0.10	0.30	0.10	0.38	0.11	0.52	0.20
	Min	3.02	0.06	0.10	0.10	0.00	0.04	0.02	0.04	0.00	0.00	0.00	0.11	0.06	0.34	0.09
	Max	3.61	0.33	0.10	0.30	0.20	0.33	0.33	0.41	0.20	0.40	0.30	0.53	0.16	0.75	0.30
**fHb ^1b^** **(g/L)**																
	Media	0.16	0.02	0.20	0.00	0.10	0.06	0.18	0.09	0.20	0.20	0.20	0.26	0.19	0.45	0.25
	Min	0.08	0.01	0.00	0.00	0.00	0.04	0.04	0.06	0.10	0.10	0.10	0.17	0.07	0.30	0.10
	Max	0.24	0.05	0.40	0.10	0.40	0.07	0.53	0.14	0.40	0.20	0.50	0.36	0.50	0.50	0.50

^1a^ MDA: malondialdehyde; ^1b^ fHb: free hemoglobin; ^2^ T0: 0 days; ^3^ T1: +7 days; ^4^ T2: +14 days; ^5^ T3: +21 days; ^6^ T4: +28 days; ^7^ T5: +35 days; ^8^ T6: +42 days; ^9^ NLR: Non-leukoreduced; ^10^ LR: Leukoreduced.

## Data Availability

All data are reported in archived datasets generated during the study. These data are available under request to Olimpia Barbato.

## Supplementary Materials

The following supporting information can be downloaded at: https://www.mdpi.com/article/10.3390/vetsci12090838/s1.

## Abbreviations

The following abbreviations are used in this manuscript:

MDA	Malondialdehyde
fHb	free hemoglobin
LR	leukoreduced
NLR	Non-leukoreduced
pRBC	Packed red blood cells
CPD-SAGM	Citrate-phosphate-dextrose saline-adenine-glucose-mannitol additive solution
CPD	Citrate-phosphate-dextrose
SAGM	Saline-adenine-glucose-mannitol
AVHTM	Association of Veterinary Hematology and Transfusion Medicine
TRACS	Transfusion Reaction Small Animal Consensus Statement
ROS	Reactive oxygen species
2,3-DPG	2,3-diphosphoglycerate
CPDA-1	Citrate-phosphate-dextrose-adenine-1
